# Whole proteome copy number dataset in primary mouse cortical neurons

**DOI:** 10.1016/j.dib.2023.109336

**Published:** 2023-06-23

**Authors:** Odetta Antico, Raja S. Nirujogi, Miratul M.K. Muqit

**Affiliations:** a MRC Protein Phosphorylation and Ubiquitylation Unit, School of Life Sciences, University of Dundee, Dundee, United Kingdom, DD1 5EH; b Aligning Science Across Parkinson's (ASAP) Collaborative Research Network, Chevy Chase, MD 20815, USA

**Keywords:** Copy Number Variation (CNV), Neuronal proteome, Label-free quantification, Neurodegenerative disease, Kinases, phosphatases, E3-ligases, Deubiquitinases (DUBs)

## Abstract

The functional diversity of neurons is specified through their proteome resulting in elaborate and tightly regulated protein interaction networks and signalling that regulates neuronal processes. Dysregulation of these dynamic networks in development or in adulthood lead to neurodevelopmental or neurological disorders respectively. Over the past few decades, mass spectrometry has become a powerful tool for quantifying and resolving any proteome, including complex tissues such as the brain proteome, with technological advances leading to higher levels of resolution and throughput than traditional biochemical techniques.

In this article, we provide a proteomic reference dataset that has been generated to identify proteins and quantify their level of expression in primary mouse cortical neurons. It represents a summary analysis of previously published data in (Antico et al., 2021).

Mouse cortical neurons were isolated from E16.5 C57Bl/6J mice and cultured for 21 *days in vitro* (DIV). We employed the mitochondrial uncouplers AntimycinA/Oligomycin (AO) to induce mitochondrial depolarisation that is a well-established paradigm to assess mitophagic signalling. Total lysates from mouse primary cortical neurons were subjected to label-free quantitative proteomic analysis using both data dependent acquisition (DDA) and data independent acquisition (DIA) modes. DDA proteomic analysis identified a total dataset of 9367 proteins in mouse cortical neurons and absolute abundance of proteins was calculated as copy numbers per cell. DDA dataset was also processed to generate a reference spectral library to fit in and quantify MS spectra generated in DIA mode. Quantitative DIA analysis identified more than 6000 protein groups and statistical comparison of the two analysed groups (untreated and AO-treated) revealed that the neuronal proteome was largely unchanged post mitochondrial depolarisation for 5 hours. To our knowledge, these files represent the most comprehensive DDA and DIA reference datasets of fully functional maturated mouse primary cortical neurons and serve as a valuable resource for further investigating the role of specific proteins involved in neurobiology and neurological disorders such as Alzheimer's disease (AD), Parkinson's disease (PD) and Autism Spectrum Disorders (ASD).

**Specifications Table**

**Table:** 

Subject	Biochemistry Neuroscience
Specific subject area	Proteomics Neuroscience: Cellular and Molecular
Type of data	Table Graph Figure Excel file of processed data
How the data were acquired	Thermo Scientific Orbitrap Exploris 480 mass spectrometer coupled to a Dionex 3000 RSLC nanoflow high-performance liquid chromatography system.
Data format	Raw data- Supplementary table (10.5281/zenodo.8023364) Processed data tables Filtered
Description of data collection	Trypsin digested proteins from primary mouse cortical neuronal cultures were fractionated using high-pH RPLC fractionation (45 fractions) and used for data-dependent acquisition (DDA) analysis. The DDA data were also used to generate a high-quality spectral library using Spectronaut version 15 (Biognosys) pulsar search engine. This spectral library was used for Data independent acquisition (DIA) proteomic analysis. The detailed protocol was previously described in dx.doi.org/10.17504/protocols.io.bs3tngnn, dx.doi.org/10.17504/protocols.io.busynwfw and (Nirujogi et al., 2021).
Data source location	MRC Protein Phosphorylation and Ubiquitylation Unit, School of Life Sciences, University of Dundee, Dundee, United Kingdom.
Data accessibility	Mass spectrometry data can be accessed either with this article or via the PRIDE partner repository (ProteomeXchange Consortium) with the dataset identifier PXD027614. https://www.ebi.ac.uk/pride/archive/projects/PXD027614
Related research article	Antico O, Ordureau A, Stevens M, Singh F, Nirujogi RS, Gierlinski M, Barini E, Rickwood ML, Prescott A, Toth R, Ganley IG, Harper JW, Muqit MMK. Global ubiquitylation analysis of mitochondria in primary neurons identifies endogenous Parkin targets following activation of PINK1. Sci Adv. 2021 Nov 12;7(46):eabj0722. doi: 10.1126/sciadv.abj0722.

## 1 Value of the Data

• This data presents a valuable and in-depth resource of the mouse primary cortical neuronal proteome and allows for the identification of proteins expressed in terminally differentiated neurons.
• The data provides absolute copy number quantification of core neuronal proteins as well as major signalling pathways associated with neurodegenerative disorders.
• This data represents a comprehensive catalogue of mouse neuronal proteins that can be interrogated by researchers to identify proteins of interest in a neuronal context and their relative amount of expression and to further examine the network complexity of molecular pathways involved in neuronal signalling and/or pivotal mechanisms linked to neuronal stress.
• This proteomic data can be used to (1) check expression of targets of drug discovery and encourage pilot hypothesis-driven studies in primary neurons; (2) identify protein biomarkers and; (3) to study neuronal pathways in physiological conditions and pathological disorders.
• This data also determines protein concentration at endogenous levels, whose information will be critical for understanding the stoichiometry relations between different proteins involved in same pathway or mechanism, and for studying physiological processes in vitro.

## 2 Objective

The fundamental objective was to produce a proteome-derived resource dataset that quantified expression of proteins derived from terminally differentiated mouse cortical neuronal cultures.

This dataset will enable quantitative analysis of protein expression in mouse cortical neurons and allow comparative analysis with different cell types derived from other cultured primary cells.

This deep proteomic analysis of mouse cortical neurons was undertaken as part of a project published by [1] related to Parkinson's-linked proteins, PINK1 and Parkin. The dataset provides a general resource for Parkinson's as it defines expression of other Parkinson's-encoded proteins including SNCA, PARK7/DJ1, VPS35, VPS13C, ATP13A2 and LRRK2 that will be of interest to the Parkinson's field more widely. It will also be a resource for related brain disorders including Alzheimer's disease and Autism Spectrum Disorder.

## 3 Data Description

**Fig. 1:**
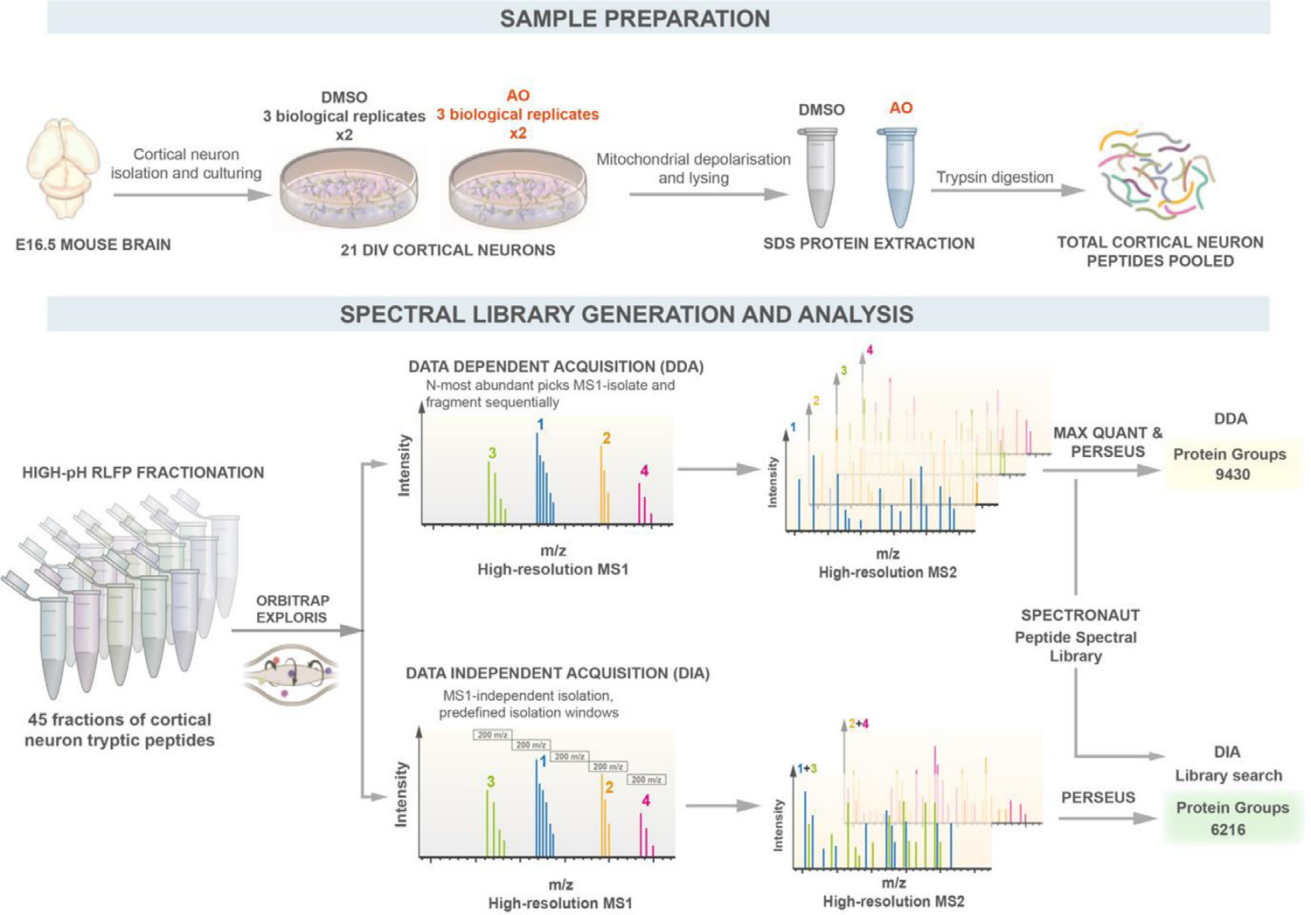
**Experimental design to quantify proteins in mouse primary cortical neurons.** Mouse cortical neurons were generated from E16.5 mouse cortices (C57BL/6J) and cultured for 21 DIV until terminal maturation. Neurons were treated with AO and DMSO for 5 hours to induce mitochondrial depolarisation (three biological replicate, in technical duplicates, for each condition). Protein lysates were prepared in SDS buffer and neuron peptides generated by trypsin digestion. Total cortical neuron peptides were fractionated in 45 fraction and subjected to LC-MS/MS analysis with Orbitrap Exploris . The neuronal proteome was analysed with 2 different acquisition modes: DDA-Data Dependent acquisition and DIA -Data independent acquisition. Spectral library was generated from DDA data and used for quantification of DIA proteins.

The data presented in this article aim to profile a dataset of expressed proteins in cultured terminal differentiated mouse cortical neurons. Mature primary neuronal cultures model the physiology of cells *in-vivo* and therefore represent a tractable system to study molecular mechanisms related to the physiological and pathophysiological functions of neuronal networks. Neuronal progenitors were isolated from E16.5 mouse cortices (C57BL/6J) and cultured for 21 days *in vitro*. To further investigate whether mitochondrial stress induces changes in the neuronal proteome, mouse cortical neurons were stimulated at 21 DIV with a combination of Antimycin/Oligomycin (10μM / 1μM) to induce mitochondrial depolarisation for 5 hours. Three biological replicates (in technical duplicate) per condition were trypsin digested using the S-Trap assisted sample preparation followed by LC-MS/MS analysis. The workflow used to isolate and culture mouse cortical neurons, for sample preparation, data acquisition and analysis is outlined in Fig. 1.

A shotgun label-free quantification (LFQ) proteomics was performed, and 45 high-pH peptide fractions were analysed on an Orbitrap Exploris 480 mass spectrometer acquired in a MS raw data were searched using MS-Fragger software suite (version 3.2) [2], identified a total dataset of 9367 proteins in mouse cortical neurons and the data was classified into several cellular subsets of proteins relevant for the biological pathways in neurons and to determine the specific level of protein abundance. Further, the data was processed using Perseus software suite [3] to calculate protein copy numbers using histone-based proteomic ruler method used to quantitatively determine protein levels by copy numbers of proteins [4].

Proteins were grouped into 10 classes based on function; subcellular localisation; and gene-risk associated-diseases (Table 1). This classification enabled us to identify 316 kinases, 209 phosphatases and 711 proteins related to ubiquitin pathways, including E3-ligases and deubiquitinases. The top 25 most abundant proteins and 25 least abundant proteins of Serine/Threonine (Ser/Thr) kinases, phosphatases, E3-ligases and deubiquitinases identified in mouse cortical neurons with their relative copy number intensity are listed in Table 2. This dataset also contained proteins involved in glycosylation (183 proteins) and metabolic pathways (1737 proteins). That dataset is also amenable to subcellular localisation expression profiling, for example we identified 403 lysosomal proteins and 931 mitochondrial proteins (Table 1), and this analysis can be extended to other organelles, such as peroxisome, Golgi apparatus and endoplasmic reticulum.

**Table 1:** **Protein classification identified in mouse cortical neurons.** Proteins were classified based on Uniprot annotation and literature references reported in the table. Alzheimer's disease (AD), Parkinson's disease (PD) and Autism Spectrum Disorder (ASD).

Classification	Number of proteins	References
Kinases	316	[5,6]
Phosphatases	209	[7]
Ub components	711	[8,9]
Glycosylation	183	[10]
Metabolism	1737	https://metabolicatlas.org
Lysosomes	403	[11]
Mitochondria	931	[12]
AD	47	[13]
PD	22	[14]
ASD	85	[15]

**Table 2:** **The most abundant and least abundant proteins in mouse cortical neurons.** Neuronal proteins were classified and searched for serine/threonine (Ser/Thr) kinases, phosphatase, E3-ligases and Deubiquitinases. The 25 most abundant and least abundant proteins for each category is reported with their relative copy number intensity. The full list can be searched in the Supplementary table.

Top 25 proteins							
Ser/Thr Kinases		Phosphatases		E3-ligases		Deubiquitinases	
Gene Names	Copy number Intensity	Gene Names	Copy number Intensity	Gene Names	Copy number Intensity	Gene Names	Copy number Intensity
**Camk2a**	3317842.58	**Pgam1**	1798634.37	**Cacybp**	515547,10	**Uchl1**	5714448.04
**Camk2d**	3185173.61	**Ppp2r1a**	854035.26	**Skp1**	511569,95	**Otub1**	785369.54
**Camk2b**	2969601.09	**Pgam2**	802371.74	**Prpf19**	179958,67	**Uchl3**	321454.91
**Camk2g**	2616152.52	**Ppp3ca**	760020.84	**Rbx1**	169664,79	**Cops6**	243962.68
**Prkaca**	1071633.62	**Ppp2cb**	717862.45	**Trim28**	126308,80	**Ufc1**	229077.67
**Prkacb**	1068027.11	**Ppp2ca**	717197.17	**Trim2**	97168,579	**Psmd14**	207401.28
**Mapk1**	1008711.93	**Ppp1cc**	667396.53	**Marchf5**	92901,443	**Eif3f**	172908.88
**Mapk3**	801052.76	**Ppp1cb**	657960.61	**Rnf146**	84911,11	**Psmd7**	135499.18
**Nek6**	600971.10	**Ppp1ca**	657511.81	**Fbxo2**	82398,91	**Usp5**	130045.04
**Map2k1**	335619.99	**Ppp3cb**	650055.00	**Stub1**	77661,35	**Cops5**	129067.14
**Csnk2a2**	332256.56	**Ppp3r1**	634752.90	**Fbxl16**	75091,29	**Trim28**	126308.80
**Csnk2a1**	312767.91	**Nudt3**	576152.38	**Trim3**	73430,40	**Eif3h**	118747.56
**Mapk9**	304336.97	**Ppa1**	569447.94	**Ppp1r11**	52708,60	**Usp14**	111636.93
**Camkv**	302724.87	**Acp1**	449663.94	**Rnf14**	50471,25	**Uchl5**	94074.43
**Gsk3b**	296476.99	**Ppp3cc**	410028.63	**Nedd4**	48190,53	**Otub2**	48130.60
**Cdk9**	246610.74	**Dusp3**	309834.65	**Sarm1**	43719,05	**Usp10**	35708.09
**Cdk5**	244572.26	**Nudt10**	307062.25	**Ddb1**	42772,67	**Usp46**	29426.69
**Prkcg**	229662.64	**Impa1**	304598.61	**Cul3**	42568,15	**Otud6b**	29155.23
**Nlk**	177133.97	**Pgam5**	283464.87	**Faf2**	42316,56	**Stambp**	22022.21
**Mapk8**	173965.76	**Itpa**	273085.32	**Rnf7**	42154,63	**Usp12**	20836.70
**Gsk3a**	169992.14	**Ppp1r7**	269556.07	**Ube3a**	42103,70	**Usp7**	18973.79
**Map2k2**	154213.12	**Nudt4**	255477.27	**Pex10**	37815,97	**Usp9x**	16536.66
**Src**	144561.37	**Ppp2r1b**	252342.32	**Trim9**	35129,50	**Usp15**	15292.21
**Pak3**	143445.85	**Nudt2**	237527.98	**Cul2**	32826,63	**Usp39**	15231.95
**Pak2**	137970.30	**Set**	221122.73	**Cul5**	27509,64	**Mindy4**	14679.13

Bottom 25 proteins							

**Irak4**	640,29	**Impa2**	1950,11	**Trim47**	471,76	**Usp54**	2728,53
**Mertk**	629,86	**Ssh1**	1942,22	**Paxip1**	444,25	**Otud4**	2456,73
**Bub1b**	583,06	**Ppp1r16b**	1935,67	**Rnft2**	403,76	**Usp48**	2382,84
**Ryk**	534,16	**Inpp4b**	1673,33	**Rnft1**	403,75	**Josd2**	2326,45
**Map4k2**	506,56	**Ptpru**	1659,44	**Neurl2**	388,90	**Rcbtb2**	2315,90
**Trpm7**	481,88	**Phlpp2**	1567,44	**Trim65**	365,57	**Otud5**	1829,44
**Pdik1l**	481,17	**Ptpn21**	1477,70	**Klhl24**	356,77	**Bap1**	1720,80
**Tgfbr1**	379,72	**Dusp8**	1345,19	**Trim56**	337,62	**Usp33**	1659,73
**Atm**	378,14	**Dolpp1**	1041,55	**Polk**	313,09	**Usp29**	1650,98
**Syk**	357,09	**Ppm1d**	876,07	**Znf511**	301,15	**Usp2**	1439,60
**Fgfr2**	303,05	**Pxylp1**	821,37	**Det1**	294,77	**Usp38**	1308,99
**Chek2**	284,87	**Rpap2**	786,93	**Rnf145**	279,54	**Mpnd**	1128,21
**Stk40**	247,21	**Ppp1r3c**	757,13	**Znf451**	261,82	**Usp34**	959,06
**Tyk2**	226,50	**Ptprm**	742,88	**Marchf7**	209,61	**Usp45**	898,21
**Hunk**	203,71	**Ptpn3**	741,15	**Rnf213**	192,56	**Usp28**	897,82
**Prkdc**	191,03	**Phlpp1**	730,99	**Map3k1**	175,31	**Usp43**	801,00
**Stk32a**	189,66	**Styx**	719,67	**Rmnd5b**	167,83	**Otud3**	744,85
**Map3k1**	175,30	**Dusp7**	690,11	**Kmt2d**	159,43	**Usp21**	610,33
**Wee1**	170,25	**Lpin1**	687,44	**Rnf168**	154,86	**Usp16**	497,08
**Eif2ak1**	161,93	**Inpp5e**	626,24	**Mpg**	117,60	**Usp6nl**	437,68
**Atr**	161,92	**Ppef1**	448,25	**Rnf180**	83,61	**Usp36**	423,22
**Ttk**	155,35	**Dusp11**	413,15	**Rnf217**	55,52	**Usp42**	251,36
**Lrrk2**	133,03	**Pon1**	151,14	**Marchf11**	46,95	**Mysm1**	225,42
**Hipk1**	93,96	**Ptpn13**	118,74	**Trim39**	41,32	**Usp40**	214,33
**Pask**	41,91	**Eya4**	64,59	**Znf521**	7,37	**Zranb1**	191,56

This multifaceted analysis was also expanded to classify proteins linked to genetic disease risk for neurological disorders. In mouse cortical neurons, distinct sets of proteins encoded by disease risk genes included: 47 AD-related genes, 22 PD-related genes and 85 ASD-related genes (Table 1). The top 15 proteins encoded by genes for each disease were reported with their relative copy number intensity and concentration (Table 3).

**Table 3:** **Proteins encoded by at risk genes for neurological disease identified in mouse cortical neurons.** The top 15 proteins for each disease were described and reported with relative copy number intensity and concentration. Alzheimer's disease (AD), Parkinson's disease (PD) and Autism Spectrum disorder (ASD). The full list of gene risk associated-proteins can be found in the Supplementary table.

	UNIPROT ID	Risk-genes	Description	Copy number Intensity	Concentration [nM]
Alzheimer's disease (AD)	P10637	Mapt	Microtubule-associated protein tau	931561.53	2972.99
	P08226	Apoe	Apolipoprotein E	807580.03	2577.31
	P17665	Cox7c	Cytochrome c oxidase subunit 7C, mitochondrial	591958.26	1889.18
	P10605	Ctsb	Cathepsin B	381539.17	1217.65
	Q7M6Y3	Picalm	Phosphatidylinositol-binding clathrin assembly protein	199646.30	637.15
	O08539	Bin1	Myc box-dependent-interacting protein 1	104298.26	332.88
	Q9WV80	Snx1	Sorting nexin-1	51377.50	163.97
	P60060	Sec61g	Protein transport protein Sec61 subunit gamma	50420.77	160.91
	G5E8K5	Ank3	Ankyrin-3	45591.86	145.50
	Q06890	Clu	Clusterin	39516.43	126.11
	P12023	App	Amyloid-beta A4 protein	39239,99	125,23
	Q8CIB5	Fermt2	Fermitin family homolog 2	39191,99	125,08
	O55033	Nck2	Cytoplasmic protein NCK2	26043,71	83,12
	P97411	Ica1	Islet cell autoantigen 1	25819,95	82,40
	Q80 × 71	Tmem106b	Transmembrane protein 106B	25286,44	80,70

Parkinson's disease (PD)	Q9R0P9	Uchl1	Ubiquitin carboxyl-terminal hydrolase isozyme L1	5714448.04	18237.12
	O55042	Snca	Alpha-synuclein	4734736.75	15110.47
	Q9EQH3	Vps35	Vacuolar protein sorting-associated protein 35	173786.37	554.62
	Q9JIY5	Htra2	Serine protease HTRA2, mitochondrial	101714.09	324.61
	Q9D1L0	Chchd2	Coiled-coil-helix-coiled-coil-helix domain-containing protein 2	82844.20	264.39
	Q8CHC4	Synj1	Synaptojanin-1	55322.43	176.56
	P17439	Gba	Lysosomal acid glucosylceramidase	48017.66	153.24
	Q80TZ3	Dnajc6	Putative tyrosine-protein phosphatase auxilin	47401.40	151.28
	Q6NZJ6	Eif4g1	Eukaryotic translation initiation factor 4 gamma 1	40337.90	128.73
	P50428	Arsa	Arylsulfatase A	28139.97	89.81
	Q8CIB6	Tmem230	Transmembrane protein 230	21644,46	69,08
	Q04519	Smpd1	Sphingomyelin phosphodiesterase	20828,19	66,47
	Q9WVS6	Prkn	E3 ubiquitin-protein ligase parkin	18235,23	58,20
	Q99KY4	Gak	Cyclin-G-associated kinase	9020,20	28,79
	Q3U7U3	Fbxo7	F-box only protein 7	6536,53	20,86

Autism Spectrum Disorder (ASD)	O08553	Dpysl2	Dihydropyrimidinase-related protein 2	5676905.37	18117.31
	B2RSH2	Gnai1	Guanine nucleotide-binding protein G(i) subunit alpha-1	3360566.06	10724.93
	O08599	Stxbp1	Syntaxin-binding protein 1	1544436.05	4928.92
	P62743	Ap2s1	AP-2 complex subunit sigma	606823.54	1936.62
	O89053	Coro1a	Coronin-1A	435622.39	1390.24
	Q60900	Elavl3	ELAV-like protein 3	294940.38	941.27
	P03995	Ctnnb1	Catenin beta-1	139727.82	445.93
	Q02248	Map1a	Microtubule-associated protein 1A	133208.60	425.12
	Q9QYR6	Gria2	Glutamate receptor 2	115444.02	368.43
	P23819	Slc6a1	Sodium- and chloride-dependent GABA transporter 1	108231.86	345.41
	Q60676	Ppp5c	Serine/threonine-protein phosphatase 5	91262,77	291,26
	P63080	Gabrb3	Gamma-aminobutyric acid receptor subunit beta-3	85522,87	272,94
	P0DI97	Nrxn1	Neurexin-1-beta	83346,00	265,99
	Q9Z1D1	Eif3g	Eukaryotic translation initiation factor 3 subunit G	69047,20	220,36
	Q9JHU4	Dync1h1	Cytoplasmic dynein 1 heavy chain 1	64145,40	204,71

**Fig. 2:**
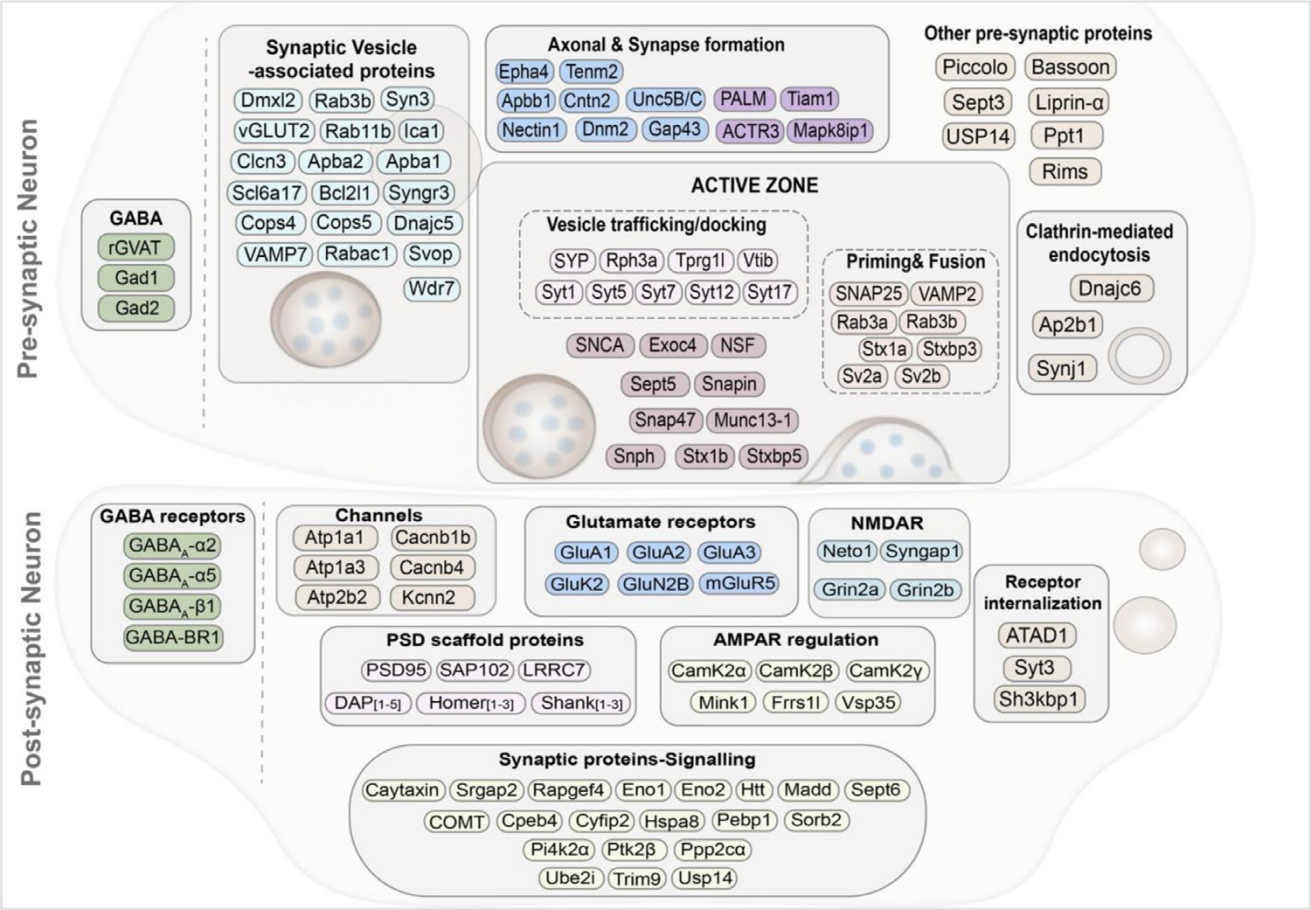
**Snapshot of synaptic proteins identified in mouse cortical neurons.** Representative image of clustering synaptic proteins found in mature primary cortical neurons. Proteins were categorised based on their subcellular localisation and role in neurotransmission.

This dataset also enables proteomic snapshots of neuronal processes. As neurons have a high compartmentalized signalling network and synaptic transmission is one of the key neuronal processes, a systematic examination was carried out for synaptic proteins. The most representative synaptic proteins identified in this dataset were clustered in the different stages of neurotransmission and shown in Fig. 2. The dataset also enabled analysis of the stoichiometry and the reciprocal proportion of the synaptic proteins connected in the same pathway or phase of neuronal process. This analysis can be extended to others cellular processes, such as neuronal metabolism and neuronal protein turnover.

A DDA spectral library was also generated from 45 high-pH fractions of mouse cortical neuronal extracts using MS-Fragger search algorithm. In parallel, sample fractions were acquired and analysed in Data-Independent acquisition mode (DIA acquisition scheme reported in Supplementary table). DIA spectra were identified and quantified using the spectral library generated from DDA data.

## 4 Experimental Design, Materials and Methods

### 4.1 Mouse cortical neuronal preparation from C57BL/6J mice

The C57BL/6J (RRID:IMSR_JAX:000664) mice were obtained from Charles River Laboratories (Kent-UK) and housed in a pathogen–free facility with temperature-controlled rooms at 21°C and 45 to 65% relative humidity, 12-hour light/12-hour dark cycles and supplied food and water *ad libitum*.

A detailed protocol describing the preparation of primary cortical mouse neurons has been published (http://dx.doi.org/10.17504/protocols.io.bswanfae) . In brief, cortices were isolated from E16.5 embryos and tissue digestion was performed by incubation with trypsin-EDTA at 37°C for 30 min. Cortical neurons were plated at a density of 5.0 × 10^5^ cells per well on poly-L-Lysine coated six-well plates and cultured in neuronal media: Neurobasal medium, 1X B27 supplement, 1X GlutaMAX, and 1X penicillin-streptomycin. Neurons were cultured in a water-saturated incubator at 37°C and 5% CO_2_ for 21 days and medium was partially replaced every 5 days for 1/3 of the total volume.

### 4.2 Sample preparation for copy number proteomics

Technical duplicates of three biological replicates of mouse neurons were treated for 5 hours with 10 μM Antimycin A and 1 μM oligomycin (AO) in DMSO at 37°C (n = 6 AO and n = 6 DMSO). Cells were lysed in buffer containing Tris-HCl (10 mM, pH 8.0), SDS (2%, w/v), sodium orthovanadate (1 mM), sodium glycerophosphate (10 mM), sodium fluoride (50 mM), sodium pyrophosphate (5 mM), protease inhibitor cocktail, and microcystin-LR (1 μg/ml). Lysates were boiled for 10 min at 95°C and then sonicated using Bioruptor for 10 min (30-s on and 30-s off, 10 cycles) at 4°C. Samples were centrifuged at 20,000 x g for 20 min at 4°C. Supernatants were collected and protein concentration was determined by using the BCA kit (Pierce).

The mass spectrometry workflow and methods used in this study, are described in detail in dx.doi.org/10.17504/protocols.io.bs3tngnn, dx.doi.org/10.17504/protocols.io.busynwfw and [16]. Briefly, an S-Trap protocol was used for either 50 μg of protein for each single AO- and DMSO-treated sample or 300 μg of pooled cortical neurons. Briefly, sample reduction was performed with 10 mM TCEP and alkylation with 40 mM Iodoacetamide. Samples were loaded on S-Trap micro and mini columns and purified by washing with S-Trap wash buffer [100 mM TEABC (pH 7.2) in 90% methanol] four times. Peptide digestion was done using Lys-C ± trypsin at 1:20 ratio and incubated at 47°C for 1.2 hours following overnight incubation at room temperature. Peptides were sequentially eluted using 50 mM TEABC buffer, 0.15% formic acid (v/v), and 80% acetonitrile (ACN) in 0.15% formic acid (v/v) and vacuum-dried. For the pooled cortical neuron, peptides generated after trypsin digestion, were subjected to high-pH RPLC fractionation to generate 45 fractions and used for data-dependent acquisition (DDA) analysis. AO- and DMSO-treated samples were dissolved in LC buffer [3% ACN in 0.1% formic acid (v/v)], and 2 μg of peptide amount was injected for DIA analysis.

### 4.3 Copy number total proteomic analysis using DDA and DIA

Copy number analysis and DDA method are described in [4]. Orbitrap Exploris 480 mass spectrometer coupled in line with Dionex 3000 RSLC nano liquid chromatography (LC) system was used to analyse the 45 high-pH fractions. Sample was injected onto trap column (Acclaim PepMap 2 cm, 3 μm particle) and separated on a 50-cm analytical column at 300 nl/ min (ES803; 50 cm, C18 2μm particle) and directly electrosprayed into the mass spectrometer using EASY nanoLC source.

Data were acquired in a DDA mode by acquiring full MS at 60,000 resolution at a mass/charge ratio (m/z) of 200 and analyzed using Orbitrap mass analyzer. MS2 data were acquired at top speed for 2s to acquire as many data-dependent scans by using 1.2-Da isolation window using quadrupole mass filter and fragmented using normalized 30% high-energy collision-induced dissociation (HCD); the MS fragment ion was measured at 15,000 resolution at 200 m/z using Orbitrap mass analyzer. Automatic gain control (AGC) targets for MS1 were set at 300% and MS2 at 100% with a maximum ion-injection accumulation time at 25 and 80 ms, respectively.

For the DIA analysis, peptide amount from each of the AO-treated and DMSO-treated cortical neuron samples were acquired on an Orbitrap Exploris 480 mass spectrometer. Peptides were loaded on trap column and eluted on an analytical column by using a nonlinear gradient of 120 min and a total of 145-min run. MS1 data were acquired at 120,000 resolution at 200 m/z and measured using Orbitrap mass analyzer. Variable DIA scheme was used by using a Quadrupole mass filter in the mass range of 400 to 1500 m/z. A total of 45 variable isolation windows employed per duty cycle and peptide precursor ions were fragmented using a normalized steeped HCD collision energy (26, 28, and 30) and measured at 30,000 resolution at m/z of 200 using Orbitrap mass analyzer. AGC targets for MS1 were set at 300% and for MS2 at 3000% with a maximum ion-injection accumulation time of 25 and 80 ms, respectively. The completed variable DIA window schemes and instrument settings are provided in the Supplementary table and have been deposited at Zenodo (doi:10.5281/zenodo.8023364).

### 4.4 Mass spectrometry data analysis

DDA raw MS data were processed using Frag pipe software suite (version 15.0- https://fragpipe.nesvilab.org/) using an in-built MS-Fragger search algorithm (version 3.2) [2,17]. Default closed search workflow was used and searched against Mouse UniProt database (2021-03-18-decoys-reviewed-contam-UP000000589.fas-https://ftp.uniprot.org/pub/databases/uniprot/previous_major_releases/release-2021_03/). Precursor mass tolerance was set at −50 and ±50 ppm (parts per million), and fragment mass tolerance was set at 20 ppm.

MS1 quantification was performed using MS-Fragger version 3.2 with an in-built IonQuant algorithm (https://github.com/Nesvilab/IonQuant; doi: 10.5281/zenodo.8098825) by allowing match between runs. One percent false discovery rate (FDR) at peptide-spectrum match (PSM), peptide, and protein level was applied for the final output files. Protein group table was further processed using Perseus software suite (v1.6.15.0- http://www.perseus-framework.org - RRID:SCR_015753) to estimate copy numbers using histone proteomic ruler [3,4]. The DDA data were used to generate a spectral library using Spectronaut version 15 (Biognosys - https://biognosys.com/software/spectronaut/)  pulsar search engine [18]. This library was used for the library-based search for DIA data by using the default search parameters and enabling cross-run normalization. The search output protein group table was exported and processed using Perseus for further analysis. Statistical analysis was completed using a Student T tests with 1% permutation-based FDR for the identification of differentially regulated proteins [3].

## Ethics Statements

All animal studies were conducted in accordance with the Animal Scientific Procedures Act (1986) and with the Directive 2010/63/EU of the European Parliament and of the Council on the protection of animals used for scientific purposes (2010, no. 63). Experiments and breeding were approved by the University of Dundee Ethical Review Committee and further subjected to approved study plans by the Named Veterinary Surgeon and Compliance Officer.

## CRediT authorship contribution statement

**Odetta Antico:** Conceptualization, Methodology, Investigation, Writing – original draft, Writing – review & editing. **Raja S. Nirujogi:** Conceptualization, Methodology, Investigation, Formal analysis, Writing – original draft, Writing – review & editing. **Miratul M.K. Muqit:** Conceptualization, Writing – original draft, Writing – review & editing, Funding acquisition.

## Declaration of Competing Interests

The authors declare the following financial interests/personal relationships which may be considered as potential competing interests:

M.M.K.M. is a member of the Scientific Advisory Board of Mitokinin Inc. and Scientific Consultant to Stealth Biotherapeutics Inc. and Merck & Co Inc.

## Data Availability

COPY NUMBER ANALYSIS OF NEURONS (Original data) (PRIDE).

## Appendix Supplementary materials

Image, application 1
